# SR-TTS: a rhyme-based end-to-end speech synthesis system

**DOI:** 10.3389/fnbot.2024.1322312

**Published:** 2024-02-27

**Authors:** Yihao Yao, Tao Liang, Rui Feng, Keke Shi, Junxiao Yu, Wei Wang, Jianqing Li

**Affiliations:** ^1^The Department of Biomedical Engineering, Nanjing Medical University, Nanjing, China; ^2^Jiangsu Province Engineering Research Center of Smart Wearable and Rehabilitation Devices, School of Biomedical Engineering and Informatics, Nanjing Medical University, Nanjing, China; ^3^Changzhou Medical Center, The Affiliated Changzhou Second People's Hospital of Nanjing Medical University, Changzhou Second People's Hospital, Nanjing Medical University, Changzhou, China

**Keywords:** text-to-speech synthesis, multilingual modeling, prosody predictor, pre-duration predictor, self-attention structure, causal convolutions

## Abstract

Deep learning has significantly advanced text-to-speech (TTS) systems. These neural network-based systems have enhanced speech synthesis quality and are increasingly vital in applications like human-computer interaction. However, conventional TTS models still face challenges, as the synthesized speeches often lack naturalness and expressiveness. Additionally, the slow inference speed, reflecting low efficiency, contributes to the reduced voice quality. This paper introduces SynthRhythm-TTS (SR-TTS), an optimized Transformer-based structure designed to enhance synthesized speech. SR-TTS not only improves phonological quality and naturalness but also accelerates the speech generation process, thereby increasing inference efficiency. SR-TTS contains an encoder, a rhythm coordinator, and a decoder. In particular, a pre-duration predictor within the cadence coordinator and a self-attention-based feature predictor work together to enhance the naturalness and articulatory accuracy of speech. In addition, the introduction of causal convolution enhances the consistency of the time series. The cross-linguistic capability of SR-TTS is validated by training it on both English and Chinese corpora. Human evaluation shows that SR-TTS outperforms existing techniques in terms of speech quality and naturalness of expression. This technology is particularly suitable for applications that require high-quality natural speech, such as intelligent assistants, speech synthesized podcasts, and human-computer interaction.

## 1 Introduction

Text-to-speech synthesis (TTS) is referred as the process of automatically generating speech waveform based on text using computer technology (Rabiner and Schafer, 2007). Essentially, it completes the unequal-length sequence mapping based on text and transfer to corresponding speech (Donahue et al., 2006). It acts like a bridge during human-computer interaction, enabling machines to communicate like real humans with understandable languages (Bharadiya, 2023). TTS technology has gone through a long history, which evolved from a rule-based synthesis (Oliviera et al., 1992) to a concatenative generation model (Lee and Cox, 2002), and then upgraded to a statistical parametric structure (Zen, 2015).

In recent years, with the rapid development of deep learning and artificial intelligence technologies, speech synthesis models based on these have achieved remarkable progress (Kumar et al., 2023). However, building high-quality TTS systems comes across many technical challenges, such as accurately capturing prosodic patterns (Teixeira, 2004), precisely controlling speech duration and pitch, as well as effectively generating different speaking tones and styles. A powerful TTS system also needs to support multiple languages, speaking styles, and accents. Current speech synthesis (Tan et al., 2021) still faces problems like poor speech quality and slow inference speed (Trang and Nguyen, 2022). Therefore, researchers put efforts into the following models to address the problems. In 2018, Wang et al. proposed the first end-to-end TTS model named Tacotron (Wang et al., 2017; Elias et al., 2021) that no longer relied on statistical parametric synthesis and improved the speech quality to some extent. However, its complex model structure resulted in a slow inference speed, which limited the feasibility in some real-time tasks. To address the low-efficiency issues from Tacotron, Ren et al. (2006, 2019) introduced the FastSpeech. Based on the Transformer structure, it allows parallel computation with a faster inference speed. Compared to Tacotron, FastSpeech also greatly improved speech quality. However, it still had poor fine-grained control over speech details. The problem that the synthesized speech lacked prosodic rhythm remained unsolved. Recently, the denoising diffusion probabilistic models (Ho et al., 2020) (DDPM) proposed by Tieleman and Hinton have shown outstanding performance in generative tasks and have been applied to speech synthesis tasks (Jeong et al., 2021). With the help of DDPM, decent improvements in speech quality can be achieved. However, its complicated computation and high time-costs block the large-scale deployment and practical applications.

To address the key issues in current speech synthesis models, particularly in terms of inference speed and the naturalness (Liu et al., 2021) of synthesized speech, this paper introduces an improved high-quality speech synthesis system based on the Transformer architecture, named SynthRhythm-TTS (SR-TTS). These issues not only affect the usability of TTS technology in dynamic human-computer interaction and real-time applications but also limit its effectiveness in multilingual and multi-style speech synthesis.

SR-TTS is an end-to-end structure designed to enhance the naturalness and efficiency of speech synthesis. It includes an encoder, a rhythmic harmonizer, and a decoder, which can precisely control the prosody, pitch, and duration of the synthesized speech, and strengthen the relationship between the input text and the corresponding audio. The integration of a duration predictor and a feature predictor further enhances the temporal consistency and rhythmic sense of the speech synthesis, while the self-attention mechanism helps the model more effectively capture the correlation between acoustic features and input text, improving the quality and naturalness of the speech.

In terms of experimental results, SR-TTS demonstrated its superiority across multiple evaluation metrics. Compared to existing TTS systems like Tacotron2 and FastSpeech2, SR-TTS showed significant improvements in speech quality, naturalness, and inference speed.

Section 2 will provide more information on the current related works. Section 3 will give a more detailed description of the model structure of the SR-TTS while Section 4 will present the experimental results of the speech evaluation and ablation studies. The last section will give a comprehensive conclusion and future work based on this study.

## 2 Related work

With the help of deep learning, research toward speech synthesis has made remarkable progress in recent years, bringing tremendous improvements in speech quality and naturalness (Holmes, 2002). This section gives a brief review of some representative works that are relevant to this paper.

Prosody is an important characteristic of speech and is responsible for rhythm and pitch variation during speech synthesis. Recent studies have focused on improving models' prosody modeling capability for better acoustic quality. Wang introduced LSTM networks (Fan et al., 2014) in Tacotron to capture prosodic features of text, which helps to generate more natural pitch contours. Kenter et al. (2019) proposed a hierarchical prosody modeling approach with contextual RNN and variational autoencoder to improve prosody prediction accuracy.

Self-attention has been widely applied in speech synthesis tasks (Yu et al., 2022) to capture dependencies between different positions in sequences (Shaw et al., 2018). Vaswani et al. (2017) first proposed the Transformer model which models long-range dependencies using self-attention and achieved state-of-the-art performance in machine translation and text modeling, resulting in an enhancement in the speech quality and accuracy. Following this paradigm, Li et al. (2019) developed the Transformer-TTS model, which builds upon the Transformer structure to produce high-quality speech, showing notable improvements over recurrent models.

Though current TTS has made significant progresses, these models still face limitations and challenges like lack of naturalness and prosody in synthesized speech (Marge et al., 2020). To address the above issues, this paper proposes the SR-TTS with a series of innovations to improve speech quality, naturalness, and controllability. In the following sections, we will elaborate on the design and experimental results of these innovations in detail.

## 3 Model

In conventional non-autoregressive speech synthesis, using only text as input is insufficient to fully predict the variance of speech, resulting in a lack of diversity and naturalness in synthesized speech. These models often cross-overfitting problems, leading to low efficiency during training. Our SR-TTS effectively addresses the one-to-many mapping problem that occurs in the traditional Transformer-based TTS and achieves highly natural and efficient speech synthesis.

### 3.1 SR-TTS overview

**Figure 1:**
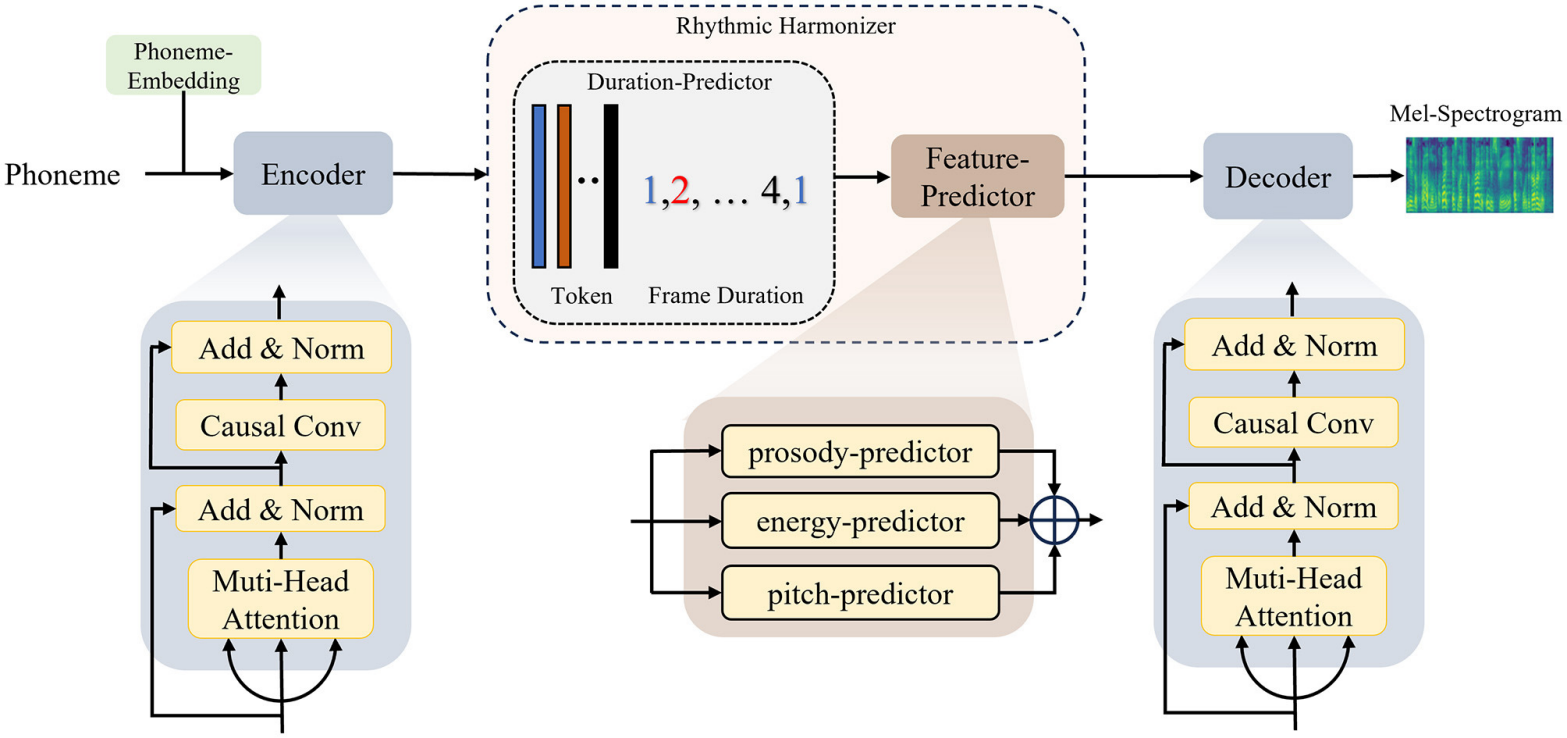
The schematic diagram of the SR-TTS.

The overall architecture of the SR-TTS is shown in Figure 1. It consists of three main modules, an encoder, a rhythmic harmonizer, and a decoder. Both the encoder and decoder adopt a Transformer-based structure that consists of three layers of multi-head attention and causal convolution layers (Kong et al., 2009). The encoder converts the input phoneme sequence into intermediate text embeddings which are then sent into the rhythmic harmonizer for further process. The rhythmic harmonizer mechanism is composed of duration and feature predictors. The front duration predictor helps control the length of each phoneme-based frame. Then the hidden sequence produced by the duration predictor then goes through the prosody, pitch, and energy predictors inside the feature predictor where different characteristics of the frames are captured. The weighted sums from the feature predictor result in an overall regulation of the speech rhythm. Finally, the decoder transfers the feature sequences into an 80-channel Mel-Spectrogram based on the corresponding input text. To enhance causal constraints on the time series, causal convolutions were introduced in both the encoder and decoder, which has greatly improved the audio generation efficiency.

### 3.2 Rhythmic harmonizer

**Figure 2:**
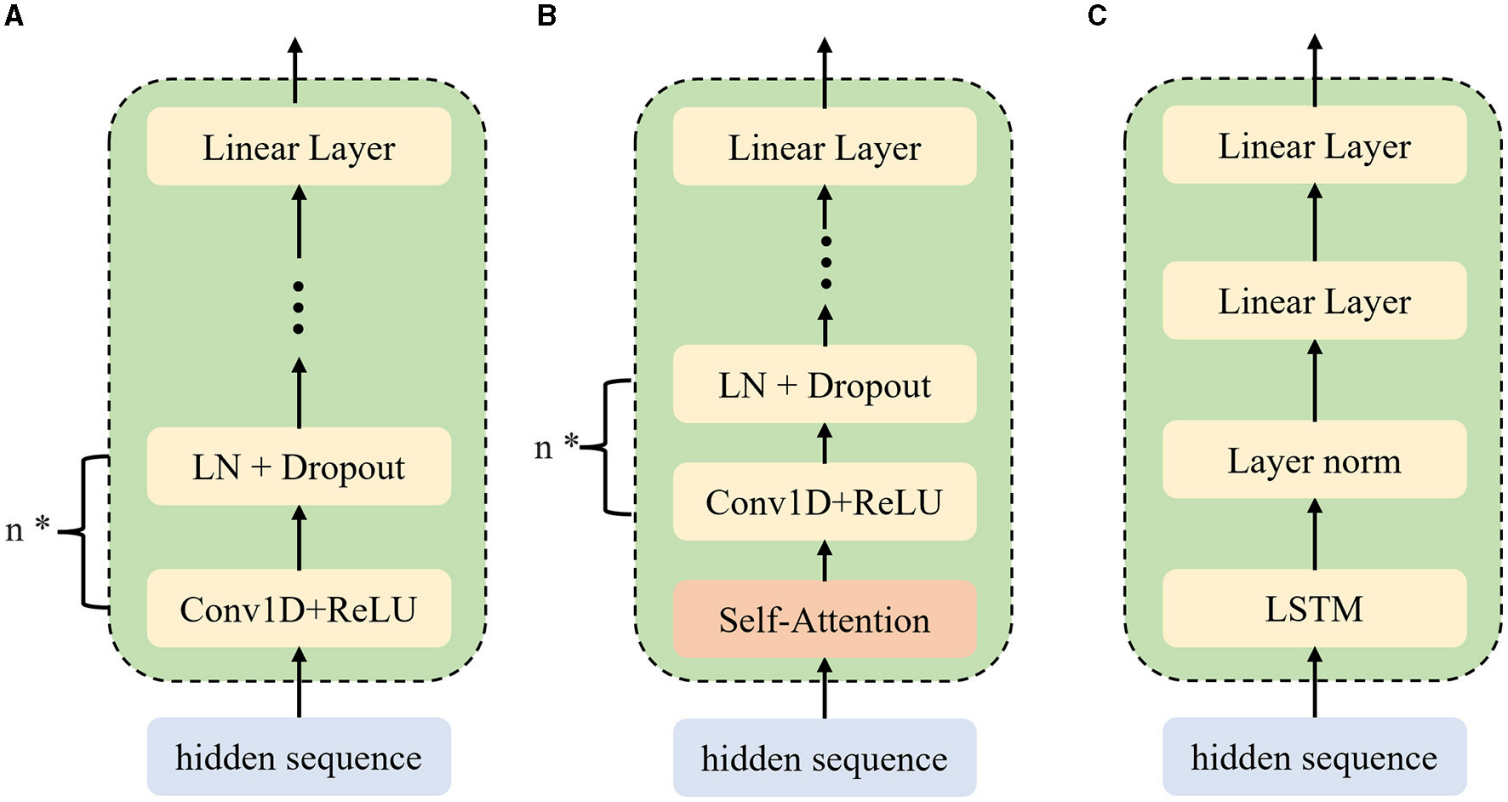
Detailed model structure of the rhythmic harmonizer. **(A)** Duration-predictor. **(B)** Energy/pitch-predictor. **(C)** Prosody-predictor. ^*^Show that this segment of the network structure is repeated *n* times.

The proposed module involves three parts which are listed below as shown in Figure 2.

#### 3.2.1 Pre-duration predictor

The front duration predictor consists of two 1D-convolution layers and one linear layer, which enables more precise control over the syllable durations as well as rhythmic sense during speech synthesis. Once the hidden representations of the input text that have been generated from the encoder are fed into the duration predictor, textual features will be extracted frame by frame. By predicting the time span of each phoneme, the duration predictor can generate and output the corresponding duration labels. By prepositioning the duration predictor at the front of the rhythmic harmonizer, the SR-TTS is able to regulate syllable durations more accurately, thereby refining temporal consistency and rhythmic sense of speech synthesis.

#### 3.2.2 Prosody predictor

Prosody is known as a combination of rhythm, pitch, and intonation of an audio signal, reflecting special rhythmic sense, pitch changes, and intonation patterns for individual speakers. As a novel module in SR-TTS, the prosody predictor consists of an LSTM net and two linear layers, which are responsible for collecting prosodic information of the input text, such as syllable boundaries and stress positions. After the hidden sequences are encoded based on the input text, they are transmitted into the LSTM net, followed by a linear layer and processed frame-by-frame to get the textual information. The textual information that carries content-based embeddings were outputted from the two linear layers and then mapped for the use of syllable boundaries and stress positions prediction. For each audio frame, the prosody predictor will also generate a prosody label to distinguish the frame between syllable boundaries and stress positions. Prosody predictor enables the model for a better capture toward the prosodic structure based on the text, thereby improving the naturalness and fluency of synthesized speech.

#### 3.2.3 Pitch and energy predictors

Pitch indicates fundamental frequency variations in speech, reflecting either high or low tone characteristics of a speaker's voice, including tones, pitch contours, and intonation patterns, which remarkably reflect emotional features in speech. Energy refers to the intensity or energy level of a speech signal and is often used to reflect the speaker's voice strength. Energy variations in speech can convey a speaker's emotional state, tone, and the manner of expression. Self-attention mechanisms are incorporated into the pitch and energy predictors to enhance modeling capabilities on pitch and energy. Continuous fundamental frequency sequences are decomposed into pitch spectrograms using continuous wavelet transform (Suni et al., 2013). The pitch spectrograms are then used as training targets for the pitch predictor and to optimize the mean square error (MSE) loss. Self-attention can capture dependencies between different positions in sequences and extract acoustic features to predict pitch and energy features of the audio, which enables the model to better capture correlations between pitch/energy, thereby improving the efficiency and accuracy of the speech synthesis.

### 3.3 Loss function

The loss function that guides the training process of the SR-TTS is a sum of the phoneme reconstruction loss, Mel-Spectrogram loss, and frame-level acoustic feature loss.

1. Phoneme Reconstruction Loss:

Phoneme reconstruction loss is introduced in the SR-TTS model to ensure that the model accurately reconstructs the input phoneme sequence and effectively captures prosodic structure changes. This loss measures the differences between the predicted phoneme probability distribution sequence and the true phoneme sequence generated by the SR-TTS. By minimizing cross-entropy loss, phoneme reconstruction loss guides the model to learn the correct influence of phonemes from prosody and duration, thereby improving the quality and naturalness of the synthesized speech. The reconstruction loss **L**_**phoneme**_ can be defined as Equation (1):


(1) $$\begin{array}{l} {\text{L}}_{\text{phoneme}}\;=\;-\frac{1}{\text{T}}\overset{\text{T}}{\underset{\text{t}=1}{\sum }}\overset{\text{N}}{\underset{\text{i}=1}{\sum }}{\text{Y}}_{\text{true}}\left(\text{t},\text{i}\right)\log\left({\text{Y}}_{\text{pred}}\left(\text{t},\text{i}\right)\right) \end{array}$$

where *Y*_*true*_ denotes the input phoneme sequence, *Y*_*pred*_ represents the predicted phoneme probability distribution, t is the sequence length, and i is the number of phonemes.

2. Mel-Spectrogram Loss:

The Mel-Spectrogram loss is calculated by the mean absolute error (MAE) loss function and measures the differences between the predicted and target Mel-Spectrograms. The Mel-Spectrogram loss **L**_**mel**_ can be defined as Equation (2):


(2) $$\begin{array}{l} {\text{L}}_{\text{mel}}\;=\;\frac{1}{\text{T}}\overset{\text{T}}{\underset{\text{t}=1}{\sum }}|\text{me}{\text{l}}_{\text{targets}\left(\text{t}\right)}-{\text{mel}}_{\text{pred}\left(\text{t}\right)}| \end{array}$$

where *mel*_*targets*(*t*)_ and *mel*_*pred*(*t*)_ are regarded as the target Mel-Spectrogram and the predicted Mel-Spectrogram, respectively.

3. Frame-Level Acoustic Feature Loss:

Self-attention mechanisms and causal convolutions are adopted in the proposed RS-TTS for an improvement in the capability of modeling acoustic features such as duration, pitch, and energy. To train the acoustic feature predictors, frame-level acoustic feature loss is implanted to measure the difference between the generated and target acoustic features. It uses Mean squared error (MSE) loss as the guidance to minimize differences between ground truth (GT) and the predicted acoustic feature, which ensures accurate capture of the target acoustic features, which advances the quality and naturalness of synthesized speech. The frame-level acoustic feature loss can be defined as Equations (3–5):


(3) $$\begin{array}{l} {\text{L}}_{\text{duration}}\;=\;\frac{1}{\text{T}}\overset{\text{T}}{\underset{\text{t}=1}{\sum }}\log\left({\text{duration}}_{\text{targets}\left(\text{t}\right)}-{\text{duration}}_{\text{pred}\left(\text{t}\right)}\right) \end{array}$$

(4) $$\begin{array}{l} {\text{L}}_{\text{energy}}\;=\;\frac{1}{\text{T}}\overset{\text{T}}{\underset{\text{t}=1}{\sum }}{\left({\text{energy}}_{\text{targets}\left(\text{t}\right)}-{\text{energy}}_{\text{pred}\left(\text{t}\right)}\right)}^{2} \end{array}$$

(5) $$\begin{array}{l} {\text{L}}_{\text{pitch}}\;=\;\frac{1}{\text{T}}\overset{\text{T}}{\underset{\text{t}=1}{\sum }}{\left({\text{pitch}}_{\text{targets}\left(\text{t}\right)}-{\text{pitch}}_{\text{pred}\left(\text{t}\right)}\right)}^{2} \end{array}$$

where *duration*_*targets*(*t*)_ and *duration*_*pred*(*t*)_ are the target and predicted duration features, *energy*_*targets*(*t*)_ and *energy*_*pred*(*t*)_ are the target and predicted energy features, *pitch*_*targets*(*t*)_ and *pitch*_*pred*(*t*)_ are the target and predicted pitch features.

The overall loss function can be represented as a weighted combination of the three loss functions comprehensively and described as Equation (6):


(6) $$L_{total}\;=\;\alpha L_{phoneme}+(1-\alpha )\left(L_{mel}+L_{duration}+L_{energy}+L_{pitch}\right)$$

where α is the hyperparameter. By minimizing the overall loss function, we can train the model to optimize phoneme reconstruction, duration control and acoustic feature generation, thereby achieve more accurate and natural speech synthesis results.

## 4 Experiments

### 4.1 Experimental setup

#### 4.1.1 Datasets

We evaluated the SR-TTS model on an English dataset LJ Speech (Ito and Johnson, 2017) and compared the synthesized speech with the mainstream models, including Tacotron2 and FastSpeech2. We also applied the proposed SR-TTS to a Mandarin Chinese dataset AISHELL3 (Shi et al., 2020) to explore its cross-lingual modeling capabilities. The LJ Speech dataset is a public speech dataset that consists of 13,100 short audio clips from a single female speaker in American English, with a total duration of 23.55 h. The AISHELL3 dataset contains 88,035 Chinese speech utterances from 218 native speakers, with a sampling rate of 22.05 kHz. Both corpus include phoneme information, speaker info, and text content.

#### 4.1.2 Environment

The proposed SR-TTS was trained with a batch size of 16, distributed on two NVIDIA GeForce RTX 3090 GPUs and each has 24GB memory to guarantee the high-quality model training. All experiments were conducted in an environment of CUDA 11.6 version, with Python 3.9 and PyTorch 1.12 + cu116. During training, GT (Ground Truth), which represents real human speech, was provided as teacher-forcing targets with the help of the Adam optimizer for weights iterative updating based on training data. Key hyperparameters include *N* = 4 for the encoder and decoder, *n* = 2 for feature predictors, 1 layer of LSTM for the Prosody Predictor, and α = 0.3.

#### 4.1.3 Evaluation

Human subjective evaluations using mean opinion score (MOS) and comparative mean opinion score (CMOS) (Shirali-Shahreza and Penn, 2018) were conducted to assess the quality and naturalness of the SR-TTS. We randomly selected 50 speech samples from those generated by each text-to-speech (TTS) system, which were then evaluated by 8 randomly selected raters. This means that for each TTS system, a separate set of 8 raters carried out the assessment, involving a total of 32 raters (8 raters per system). Specific criteria were used in the selection of raters to ensure the quality and consistency of the assessments. Each rater was a native speaker, had good listening skills and no hearing impairment, and had a normal educational background. The raters were asked to listen carefully to these speech samples and rate them on a scale of 1 to 5 based on the overall quality of the speech, intelligibility, natural fluency, and clarity of articulation, where 1 represents very poor speech quality and 5 represents excellent speech quality. We counted all the ratings of all the speech samples and calculated the MOS score following the Equation (7) below:


(7) $$\begin{array}{l} MOS\;=\;\frac{\sum_{n=1}^{N}R_{n}}{N} \end{array}$$

CMOS Experiment: 50 speech samples from each TTS were selected. Each pair of speech samples that from two different TTS systems was placed in A/B order (A is the compared model and B is the proposed SR-TTS in this paper), and eight raters were invited to score the paired speeches ranging from−3 (B is much better than A) to 3 (A is much better than B).

### 4.2 Results

#### 4.2.1 Speech synthesis ability

We evaluated the speech quality for our proposed SR-TTS model, conventional models including Tacotron2 and FastSpeech2, and GT as baseline through MOS and CMOS assessments on both LJ Speech and AISHELL3 datasets. In the experiments, 17 native listeners (including 8 native Chinese speakers and 8 native English speakers, as well as one speech quality assessment expert) were asked to rate the naturalness of synthesized speech samples, from which MOS and CMOS were calculated and represented in Table 1.

**Table 1:** Mean opinion score (MOS) evaluation with 95% confidence interval computed from the t-distribution for various systems on different datasets.

**System**	**LJ Speech**			***p*-value**	**AISHELL3**			***p*-value**
	**MOS**	**CMOS**	**Inference Time(/speech)**		**MOS**	**CMOS**	**Inference Time(/speech)**	
GT	4.38 ± 0.03	N/A	N/A	0.4305	4.31 ± 0.04	N/A	N/A	0.3501
Tacotron2	3.78 ± 0.05	−0.170	3.54s	0.1736	N/A	N/A	N/A	N/A
FastSpeech2	3.89 ± 0.08	−0.008	1.21s	0.0746	3.71 ± 0.11	−0.012	1.76s	0.0834
SR-TTS	4.10 ± 0.02	N/A	0.97s	0.5011	4.02 ± 0.04	N/A	1.54s	0.4825

To verify inter-rater agreement, we performed a statistical consistency test on the MOS scoring results. By using the ANOVA (analysis of variance) method, we assessed inter-rater agreement across systems. The results showed that for all systems (including SR-TTS, Tacotron2, FastSpeech2, and GT, inter-rater agreement was relatively high as the ANOVA *P*-values for all systems were >0.05, which indicated that there were no significant inter-rater differences. These findings support the reliability and stability of our MOS scoring results. Therefore, it is reasonable to assume that there is a degree of consistency in the raters' evaluations of the different systems, thus enhancing the credibility of our findings.

One of the main contributions of this study is the incorporation of rhythm prediction during speech synthesis to make it more natural with more expressive rhythmic patterns. We analyzed the rhythmic aspects of synthesized speech and mainly focused on speech features like speech rate, stress patterns, and intonation.

**Figure 3:**
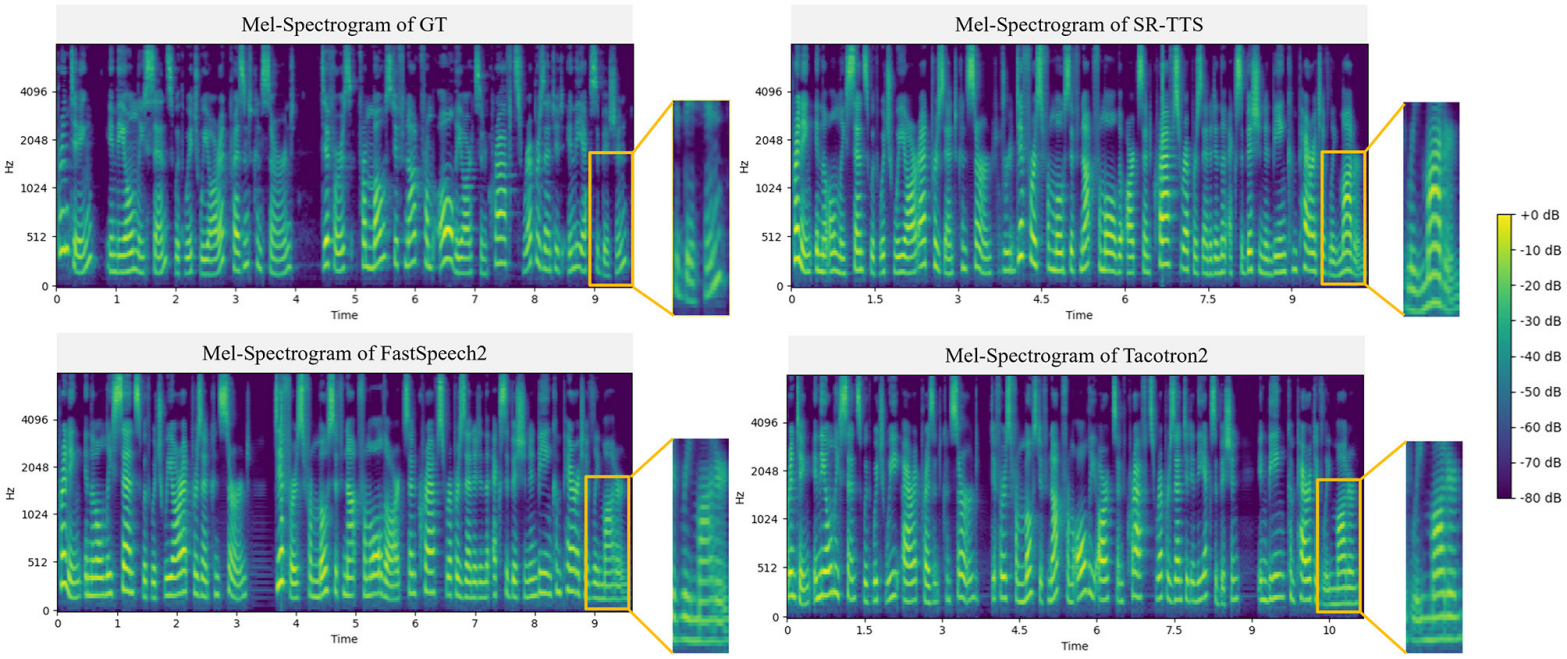
Comparison of synthesized Mel-Spectrograms based on the LJ Speech.

**Figure 4:**
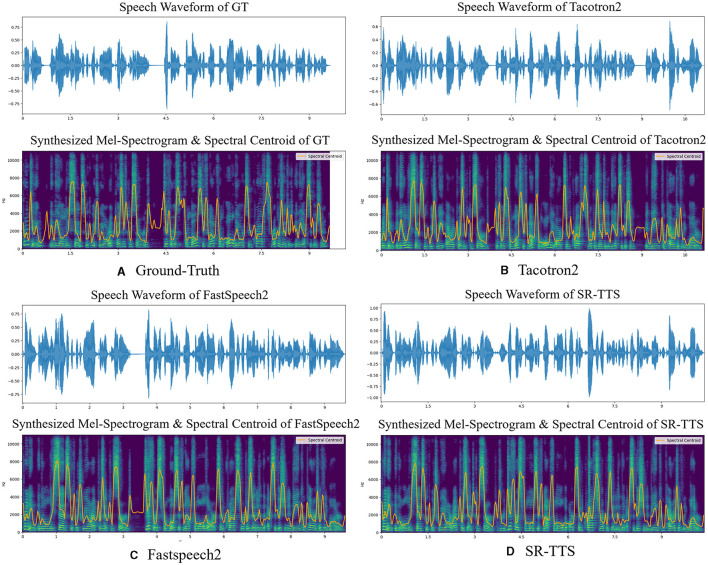
Comparison of speech waveforms and spectrograms with spectral centroid from GT and SR-TTS based on the LJ Speech.

When applying models on English dataset LJ Speech, the results from Table 1 indicate that our proposed SR-TTS is superior to the conventional TTS models as it achieved the highest MOS of 4.10 ± 0.02 compared to Tacotron2 (MOS = 3.78 ± 0.05) and FastSpeech2 (MOS = 3.89 ± 0.08), which is close to the GT (MOS = 4.38 ± 0.03). Figure 3 shows the comparison of the rhythmic features observed in Mel-Spectrograms from GT, SR-TTS, FastSpeech2 and Tacotron2 using only LJ Speech for an English speech generation comparison. The detailed Mel-Spectrograms presented in the small yellow box in Figure 3 imply that SR-TTS has advantages over innovation changes compared to the other two conventional models as it is smoothly and steadily without obvious abrupt changes or discontinuities. This also indicates the energy distribution of the speech sounds from the SR-TTS is evenly distributed without noticeable noise or distortion. At the same time, the Mel-Spectrogram shows a rich dynamic range and transitions from relatively lower energy levels to higher ones. Such dynamic range signifies that the synthesized speech from SR-TTS has good volume variation and audio detail expression capabilities. In addition, distinct rhythmic and pitch patterns manifested as periodic energy and frequency variations that reflect the rhythm, intonation, and pitch changes of the speech are obviously noticed. Such rhythmic patterns make the speech lively and naturally fluent. As shown in Figure 4, the spectral centroid (SC) parameter in the Mel-Spectrograms can be used to evaluate the luminance characteristics of speech. The experimental results show that the SC value of the speech synthesis by our proposed SR-TTS system is closer to that of a real human voice, indicating that the SR-TTS can synthesize more natural and accurate variations of speech luminance. This verifies the advantage of the SR-TTS system in capturing the spectral details of speech.

#### 4.2.2 Multilingual ability

**Figure 5:**
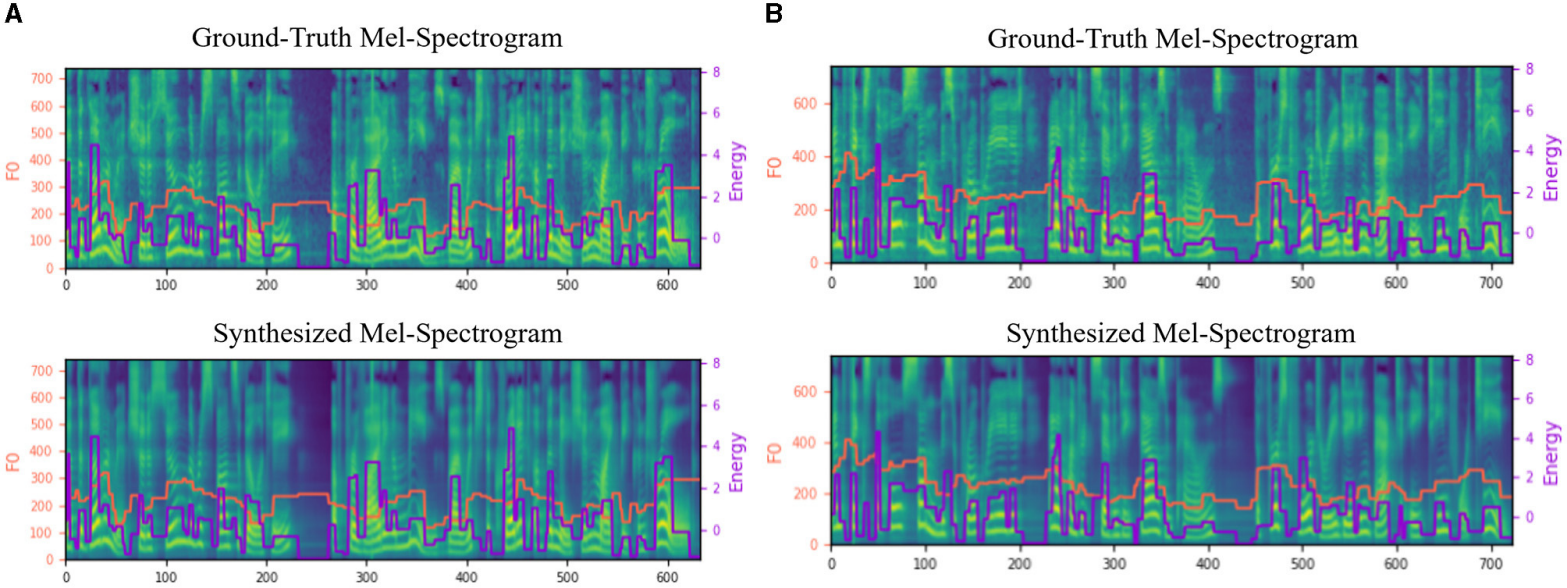
Comparison of Mel-Spectrograms for synthesized speech on **(A)** LJ Speech and **(B)** AISHELL3.

F0 is an important acoustic feature that describes the fundamental frequency of speech and corresponds to the height of the human voice. It conveys the intonation of speech by the vibration frequency of the vocal folds and is a key element in conveying semantic information. Energy describes the intensity of the speech signal, which reflects the volume and strength of the speech, and can express the emotion and tone of the speaker. Spectral patterns play a crucial role in speech synthesis as they help improve the naturalness and expressiveness of synthesized speech. Figure 5 shows the comparisons of Mel-Spectrograms generated from the SR-TTS and GT on LJ Speech and AISHELL3 for further comparisons between the synthesized and real speeches in terms of pitch contours, durations, and energy patterns. It is obvious to see that the Mel-Spectrograms shown in Figure 5 have clear pitch contour and smooth energy changes with bright colors in both English and Chinese datasets, which demonstrates the efficacy of our proposed model in capturing spectral features regardless of language.

From Table 1, the proposed SR-TTS not only achieve good performance in English dataset, but also has outstanding ability in Chinese dataset. When applying models on the Chinese dataset AISHELL3, SR-TTS achieves a MOS of 4.02 ± 0.02 in terms of speech naturalness while the GT is 4.31 ± 0.04, which still outperforms compared to the conventional FastSpeech2 (MOS = 3.71 ± 0.11), which good evidence that our SR-TTS is cable of multilingual TTS tasks.

The results of the negative CMOS indicate that the proposed SR-TTS model achieves better performance when compared to Tacotron2 and FastSpeech2 on either LJ Speech or AISHELL3, which validates the efficacy of our rhythmic and duration modeling techniques in improving synthesis speech naturalness.

### 4.3 Ablation experiments

In this section, we conducted three ablation studies on the LJ Speech dataset to evaluate the impact of the rhythm predictor module, pre-duration predictor, and self-attention mechanism on our proposed model. Moreover, we selected speech segments with different characteristics for the ablation experiments to visualize the results of the ablation experiments. The MOS evaluation results are shown in Table 2 while baseline is the original model without a rhythm predictor, pre-duration, and the self-attention.

**Table 2:** MOS evaluation with a 95% confidence interval computed from the t-distribution for the ablation study.

**Method**	**MOS**
Baseline	3.86 ± 0.12
+pre-duration	3.92 ± 0.09
+prosody-predictor	4.01 ± 0.06
+self-attention	3.89 ± 0.08

#### 4.3.1 Rhythm predictor

**Figure 6:**
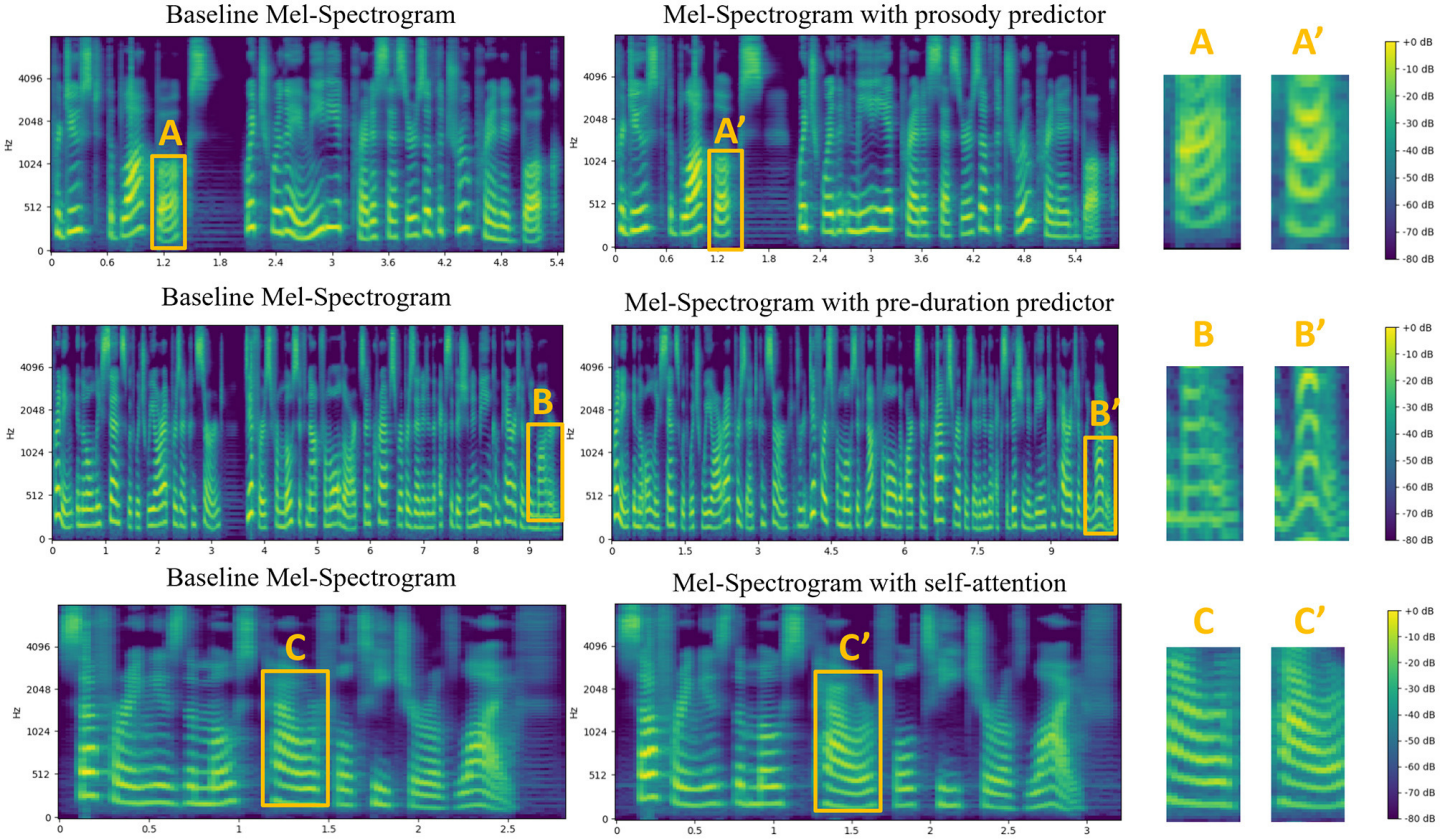
The results of the ablation studies. The **left column** is the Mel-Spectrogram of baseline without the prosody predictor, the pre-duration predictor and self-attention; The **middle column** represents the Mel-Spectrogram from the improved model with additional structures. The comparison details from each pair can be found as A and A', B and B', C and C' on the **right column**, respectively.

Figure 6 shows an intuitive comparison of the Mel-Spectrograms generated by models with or without (baseline) the rhythm predictor where a representative set of speech samples and several key differences can be observed:

(1) Rhythm patterns: Distinct rhythm patterns characterized by consistent changes in energy and spectral content occurring at fixed intervals can be obviously observed in the Mel-Spectrograms synthesized from the model with the rhythm predictor. These patterns reflect the natural prosodic features of human speech including stress, rhythm, and intonation. In contrast, the Mel-Spectrograms of the baseline lack these prominent rhythm patterns and thus sound less expressive and rhythmically constrained.
(2) Spectral smoothness: The Mel-Spectrograms of the model with the rhythm predictor demonstrate smoother transitions across frequency bands, resulting in a more continuous and natural spectrogram representation. In contrast, the Mel-Spectrograms of the baseline model exhibit more pronounced discontinuities and less smooth frequency contours, which leads to less natural-sounding speeches.

The Mel-Spectrograms of the model with the rhythm predictor exhibit more expressiveness and accurate rhythm sense with distinct rhythm patterns and smoother spectral transitions. These attributes advance the naturalness and emotional richness of the synthesized speech. The rhythm predictor plays a crucial role in capturing and generating rhythmic patterns which helps enhance the naturalness and expressiveness of synthesized speech, making it sound more fluent. Compared to the model without rhythm prediction, our model generates speech with improved quality, intelligibility, and naturalness.

#### 4.3.2 Pre-duration predictor

To explore the impact of whether preposing the duration predictor improves our speech synthesis system, we conducted comparison experiments on models with and without the preposed duration predictor. As shown in Figure 6, prepose the duration predictor has impressive effects on enhancing the smoothness and rhythm patterns of the synthesized Mel-Spectrogram. Duration predictor significantly reduces the inconsistency in time duration for each frame between synthesized Mel-Spectrogram and GT, which improves the naturalness and accuracy of rhythm. Results shown in Figure 6 reinforce the importance of the proposed duration predictor in improving the quality, naturalness, and rhythmic sense of TTS systems.

#### 4.3.3 Self-attention

To further enhance the modeling capabilities of linguistic features in SR-TTS, we introduced a self-attention mechanism in pitch and energy predictor. In ablation studies, we compared the feature prediction module with and without self-attention and analyzed the generated pitch and energy contours by inspecting the Mel-Spectrograms, the results are shown in Figure 6. From Figure 6, we found that the one with self-attention captures more subtle and smooth frequency variations. In some sentences with complex intonation, the pitch changes generated by the original model may exhibit unnatural sudden changes or discontinuities, while the model with self-attention can produce more coherent and natural pitch curves. This indicates that the self-attention mechanism enhances the model's capability in modeling pitch changes by capturing global dependencies between pitch and energy features, which also proves that self-attention effectively strengthens the model's prosody modeling capability through context modeling, thereby improving the naturalness of synthesized speech.

## 5 Discussion and conclusion

In this paper, we proposed a novel SR-TTS model that consists of a series of innovations to address the current limitations of traditional speech synthesis systems. The main structural parts of the SR-TTS are made of an encoder, a rhythmic harmonizer, and a decoder. Both the encoder and decoder inherit a Transformer-based structure so that the model can more effectively capture textual and acoustic features. Also, the replacement of regular convolutional layers with causal convolution allows for efficient processing of long sequences of data by preciously preserving the temporal order of the time-series signal in a causal fashion.

In model comparison experiments, our SR-TTS model achieves a higher MOS (Mean Opinion Score) than the traditional TTS models Tacotron2 and FastSpeech2, no matter which language it is in. In addition, the proposed SR-TTS shows great potential in real-time speech synthesis tasks with the shortest inference time among all TTS methods. Mel spectrogram analyses of LJ speech and AISHELL3 provide valuable insights into the acoustic properties of the synthesized speech, and the observed smooth and continuous spectral contours demonstrate the cross-linguistic capability of the proposed SR-TTS model to overcome language barriers. Tacotron2 and FastSpeech2 are chosen for comparison because they are among the most representative and widely used TTS models and are the current mainstream of TTS in autoregressive modeling.

The MOS evaluation results from the ablation studies indicate that the rhythmic harmonizer plays a vital important role in the overall structure of the SR-TTS. By preposing the duration predictor, the rhythmic harmonizer takes well control on the overall duration for each synthesized frame, including the total time span of the whole speech and the length of each small syllable. Compared to FastSpeech2, this novel change in our SR-TTS contributes to a more natural and expressive speech synthesis. In addition, the feature predictor that is responsible for the prediction of prosody, energy, and pitch greatly increases the accuracy of the synthesized speech, especially in terms of speaking style and pronunciation. The cooperation of the above results in significant improvements and enhancements of the model's efficiency.

In conclusion, the proposed SR-TTS model achieves good performance in enhancing speech quality and naturalness across multiple languages, while also significantly speeding up the inference process. This demonstrates its great potential for various applications in different fields. However, there are some limitations and unresolved challenges that require further exploration in the field of artificial TTS systems. One limitation is the reliance of the current deep neural network-based SR-TTS on a substantial amount of data for training, which constrains the model's effectiveness in synthesizing minority languages. Future research directions will focus on further optimizing the model architecture and hyperparameters, exploring more sophisticated rhythmic modeling methods, and enhancing the modeling capabilities for audio features. Additionally, to affirm the model's multilingual ability, there is a need to test datasets based on other languages for a more comprehensive experimental evaluation. Addressing these limitations will be crucial in advancing the capabilities and applicability of TTS systems in a wider range of languages and contexts.

## Data availability statement

The raw data supporting the conclusions of this article will be made available by the authors, without undue reservation.

## Author contributions

YY: Conceptualization, Data curation, Methodology, Software, Writing – original draft, Writing – review & editing. TL: Data curation, Validation, Writing – review & editing. RF: Formal analysis, Writing – review & editing. KS: Writing – review & editing. JY: Methodology, Visualization, Writing – original draft, Writing – review & editing, Conceptualization, Formal analysis. WW: Investigation, Methodology, Resources, Writing – review & editing. JL: Funding acquisition, Project administration, Resources, Writing – review & editing, Supervision.
